# Melanocortin Receptor Signaling Modulates Anti-Obesity and Glucose Tolerance Effects of *Hafnia alvei* 4597 Probiotic Strain in Mice

**DOI:** 10.3390/ijms27156963

**Published:** 2026-08-03

**Authors:** Vasiliy A. Zolotarev, Vladimir O. Murovets, Egor A. Sozontov, Anastasia L. Sepp, Ekaterina A. Lukina, Raisa P. Khropycheva, Benjamin Thomas, Sergueï O. Fetissov

**Affiliations:** 1 Pavlov Institute of Physiology, Russian Academy of Sciences, Saint-Petersburg 199034, Russia; murovetsvo@infran.ru (V.O.M.); egorgius@yandex.ru (E.A.S.); seppal@infran.ru (A.L.S.); lukinaea@infran.ru (E.A.L.); khropychevarp@infran.ru (R.P.K.); 2 Inserm UMR1239 Laboratory, University of Rouen Normandie, 76130 Mont-Saint-Aignan, France; benjamin.thomas12@univ-rouen.fr

**Keywords:** *Hafnia alvei*, Agouti yellow, energy metabolism, caseinolytic protease B, melanocortin receptor

## Abstract

The present study aimed to elucidate whether the metabolic effects of the probiotic bacterium *Hafnia alvei* 4597 depend on melanocortin receptor (MCR) signaling. The response to a 3-week intragastric treatment with the *H. alvei* bacterial suspension or total protein extract was compared between genetically similar mouse sub-strains but with different MCR sensitivity: KK and KK.Cg-Ay/a (KK-Ay)—the latter overproducing Agouti protein. Treatment of KK mice with *H. alvei* protein extract stimulated energy expenditure and carbohydrate oxidation but reduced lipid oxidation, leptin level, pancreatic weight, and hypothalamic insulin and leptin receptor mRNA expression. Live bacteria in KK mice reduced food intake and stimulated hypothalamic mRNA expression of proopiomelanocortin, Agouti-related peptide, and insulin receptors. In the sub-strain KK-Ay, probiotics had no effect on the aforementioned metabolic parameters. *H. alvei*-based probiotics improved glucose tolerance and decreased body fat and liver glycogen in both mouse sub-strains. Activation of MC4R by *H. alvei* protein extract was revealed by in vitro study using β-arrestin recruitment. These findings confirm beneficial effects of the *H. alvei* bacteria and show that, for several parameters, the bacterial protein extract may be more effective than bacterial suspension. Differential responses to the treatment between the mouse sub-strains, particularly in energy metabolism, as well as in vitro data, indicate that the effects of *H. alvei* are mediated by MCR signaling.

## 1. Introduction

*Hafnia alvei* is a commensal bacterium belonging to the *Hafniaceae* family (formerly the *Enterobacteriaceae* family). It is widely distributed in the digestive tracts of animals, where it produces a number of bioactive compounds [1]. This bacterium attracted attention as a representative of next-generation probiotics with a partially defined molecular mechanism of beneficial action [2]. The bacterial strain *H. alvei* 4597 has been shown to influence host metabolism by reducing food intake and body weight in both obese mice and overweight humans [3,4,5]. These beneficial anti-obesity effects were attributed to the production of the 96 kDa heat shock protein caseinolytic protease B (ClpB) by *H. alvei*, which was earlier identified in *Escherichia coli* (*E. coli*) as an antigen mimetic of α-melanocyte stimulating hormone (α-MSH), an anorexigenic neuropeptide [6] that plays a key role in the regulating energy balance [7]. It decreases food intake and increases energy expenditure through the activation of the melanocortin type 4 receptor (MC4R) [8]. Importantly, a recent study revealed that *E. coli* ClpB is also able to directly activate MC4R almost as potently as α-MSH [9].

The MC4R is expressed in numerous brain regions, with particularly high density in the paraventricular nucleus of the hypothalamus as well as in the dorsal motor nucleus of the vagus [10]. At the periphery, α-MSH exerts a direct effect on the enteroendocrine system, thereby enhancing the incretin response, inhibiting ion transport and motility, and stimulating the release of satiety factors acting on vagal afferents. MC4R mRNA has been detected in the enteroendocrine cell preparation in mice, thus confirming its status as one of the most highly expressed GPCRs in the glucagon-like peptide-1 (GLP-1)/peptide tyrosine-tyrosine (PYY)-producing L cells [11].

The anorexigenic effects of *H. alvei* resemble those activating the α-MSH/MC4R signaling. Supplementation with *H. alvei* in overweight humans undergoing a mild hypocaloric diet led to a greater loss of body weight and body fat content as well as an increase in fulness perception as compared to a placebo. Furthermore, the study revealed that administration of *H. alvei* to overweight but otherwise healthy non-diabetic subjects led to a slight yet significant decrease in basal glycemia [3].

The ClpB, its fragments, and ClpB containing protein extract from *E. coli* have been observed to reduce food intake and increase the level of PYY in mice [12,13]. Treatment of *ob*/*ob* obese prediabetic mice fed a standard diet with *H. alvei* was accompanied by decreased body weight and fat-mass gain along with reduced food intake [4]. Moreover, diet-induced obese mice treated with *H. alvei* showed a decrease in glycemia, plasma total cholesterol, and alanine aminotransferase [5]. Recently, we have shown that *H. alvei* protein extract and the recombinant *E. coli* ClpB protein were able to increase glucose tolerance in lean non-diabetic mice [14].

Although it is evident that the probiotic strain *H. alvei* 4597 is capable of improving energy metabolism in obese and diabetic rodents and overweight humans, the extent to which the favorable in vivo metabolic effects are mediated by the melanocortin receptors (MCRs) is unknown. Moreover, the ability of *H. alvei* protein extract to activate MC4R was not investigated. The present study aimed to elucidate these questions using two sub-strains of mice, Kuo Kondo—KK.Cg-a/a (KK), as well as the closely related KK.Cg-Ay/a sub-strain (KK-Ay). The inbred mouse strain KK has been employed as a polygenic model of mild obesity and T2D, exhibiting features such as glucose intolerance, hyperinsulinemia, hyperleptinemia, and polyuria [15]. This is particularly notable due to mutations of hepatic and renal esterases when compared to strains that do not manifest signs of diabetes [16]. The KK-Ay is a sub-strain derived from the KK, which was the result of the transfer of the lethal Agouti yellow obese allele (Ay). Heterozygous KK.Cg-Ay/a mice are severely obese and characterized by evident adiposity, hyperphagia, hyperglycemia, hyperinsulinemia, and glucose intolerance by the age of eight weeks [17,18]. This model is considered appropriate to study obesity and the effects of anti-diabetic drugs for T2D [18,19,20].

Agouti signaling protein (ASIP or the Agouti protein), sharing a striking similarity in structure and function with Agouti-related peptide (AgRP), is normally expressed in the skin; however, mice with the Agouti yellow lethal mutation express ASIP ubiquitously [21]. The Agouti protein is a potent competitive antagonist of the MC1R and MC4R with somewhat weaker affinity at MC3R. An earlier study reported that in mice the Agouti protein did not interact with MC3R at all [22]. Additionally, Agouti protein antagonizes adrenocorticotropic hormone (ACTH) receptor MC2R expressed in adipocytes [23]. It also inhibits the generation of cAMP stimulated by α-MSH at human MC1R and MC3-5R or by ACTH at human MC2R [24]. AgRP is a neuropeptide produced in the arcuate nucleus of the hypothalamus, inducing orexigenic effect by blocking the action of melanocortin peptides. AgRP does not antagonize the MC1R but does interact with the centrally expressed MC3R and MC4R [25,26] functioning as an inverse agonist at the constitutively active MCRs [27], expressed in hypothalamic neurons regulating feeding behavior [10,28].

Our working hypothesis was that if the effects of the *H. alvei*-based probiotic are manifested in the KK strain with intact MCR signaling but not in the KK-Ay strain having the same genotype except the Ay lethal allele, it can be assumed that the probiotic acts mainly by modulating the activity of the host MCRs. To test this hypothesis, in our proof-of-concept study, a response to 3-week intragastric treatment with a suspension of live *H. alvei* bacteria or *H. alvei* total protein extract were compared between KK and KK-Ay mice.

## 2. Results

### 2.1. H. alvei-Based Probiotics Affect Body Composition

Before starting gavage with probiotics, body weight of the KK-Ay controls was significantly higher than that of the corresponding KK controls (*p* < 0.002, *t*-test). Within each mouse strain, body weight was similar between groups: KK controls—31.46 ± 0.81 g; KK with *H. alvei* protein—30.30 ± 1.00 g; KK with *H. alvei* suspension—31.73 ± 1.15 g; KK-Ay controls—35.41 ± 0.81 g; KK-Ay with *H. alvei* protein—36.31 ± 1.06 g; and KK-Ay with *H. alvei* suspension—34.96 ± 0.72 g.

**Figure 1:**
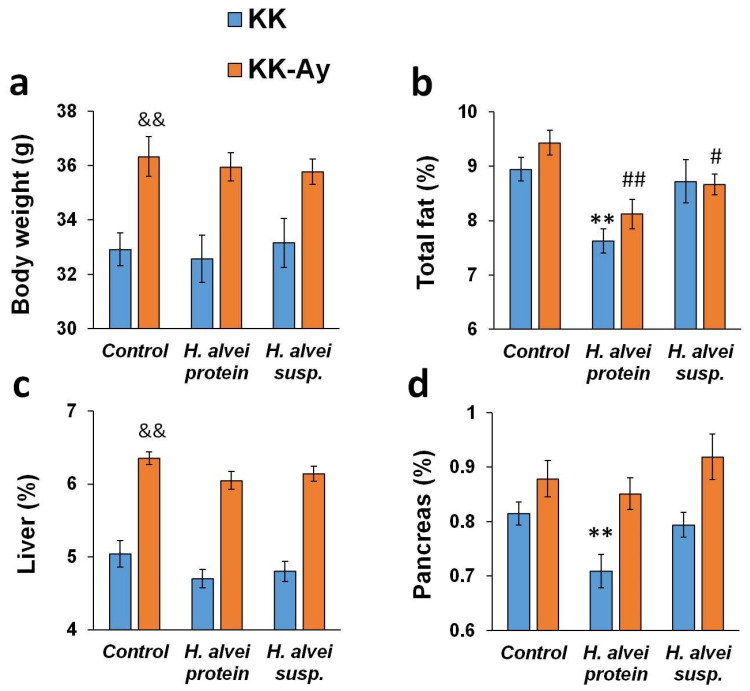
The effect of a 21-day gavage of KK.Cg-a/a (KK) and KK.Cg-Ay/a (Agouti yellow; KK-Ay) mice with total protein extract of *Hafnia alvei* (*H. alvei protein*) or suspension of *H. alvei* bacteria (*H. alvei* susp.). Body weight (**a**), body fat content (**b**), liver mass (**c**) and pancreatic mass (**d**) relative to 100% of body mass (**b**–**d**). Control mice received 200 µL of saline by gavage. Comparisons using Student’s *t*-test: && *p* < 0.01—between control groups of KK and KK-Ay; ** *p* < 0.01—KK control vs. KK protein; # *p* < 0.05, ## *p* < 0.01—KK-Ay control vs. KK-Ay experiment group. Number of animals KK/Agouti yellow per group: controls—12/15, *H. alvei* protein extract—5/10, *H. alvei* suspension—6/8.

By the end of experiment, mice from the control KK-Ay sub-strain still exhibited greater body mass and the relative mass of the liver normalized to body weight in comparison to the controls from the KK group. Treatment of KK-Ay with both probiotic preparations had no effect on these parameters (Figure 1a,c). The administration of bacterial protein extract led to a significant reduction in relative fat mass in both sub-strains (Figure 1b) and a decrease in relative pancreatic mass in the KK group (Figure 1d). 

### 2.2. Glucose, Insulin, and Leptin Levels

**Figure 2:**
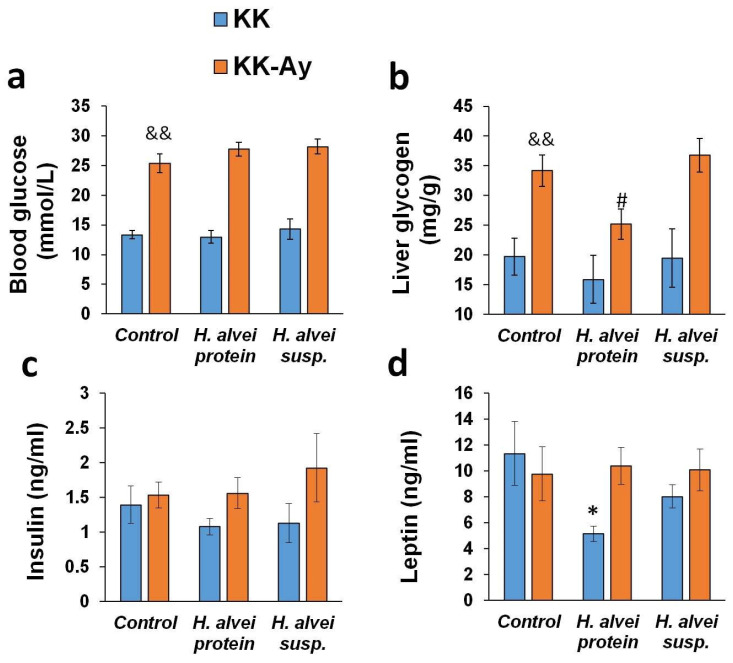
The effect of intragastric administration of *H. alvei* protein extract (*H. alvei protein*) or *H. alvei* bacterial suspension (*H. alvei* susp.) on the baseline glucose level (**a**), glycogen concentration in the liver (**b**), insulin (**c**) and leptin (**d**) levels in plasma in mouse sub-strains KK.Cg-a/a (KK) and KK.Cg-Ay/a (Agouti yellow; KK-Ay). Non-fasted animals after 21 days of probiotic treatment. Control mice received 200 µL of saline by gavage. Comparisons using Student’s *t*-test: && *p* < 0.01—between control groups of KK and KK-Ay; * *p* < 0.05—KK control vs. KK protein; # *p* < 0.05—control KK-Ay vs. KK-Ay protein group. Number of animals per strain: KK control—9~12; KK with *H. alvei* protein—4~5; KK with *H. alvei* suspension—6; KK-Ay control—11~15; KK-Ay with *H. alvei* protein—9~10; KK-Ay with *H. alvei* suspension—8.

Non-fasted plasma glucose levels were assayed on the last day of experiment before euthanasia. The control group of KK-Ay mice exhibited significant hyperglycemia with plasma glucose almost twofold higher than in the corresponding KK group (Figure 2a). No influence of probiotic treatment on glucose levels was observed in either mouse sub-strain (Figure 2a). The hepatic glycogen concentration was greater in the KK-Ay group, it was reduced after treatment with bacterial protein extract (Figure 2b). There were no significant differences in insulin levels between control and treated groups (Figure 2c). There was no difference in plasma leptin between the control groups of two mouse sub-strains, but it was reduced in KK mice treated with *H. alvei* protein extract. This trend was only observed in KK mice receiving suspension of the live bacteria *H. alvei* (Figure 2d). No effect of probiotic treatment on plasma leptin levels was observed in KK-Ay mice (Figure 2d).

### 2.3. Probiotic Treatment Shifts Energy Balance

**Figure 3:**
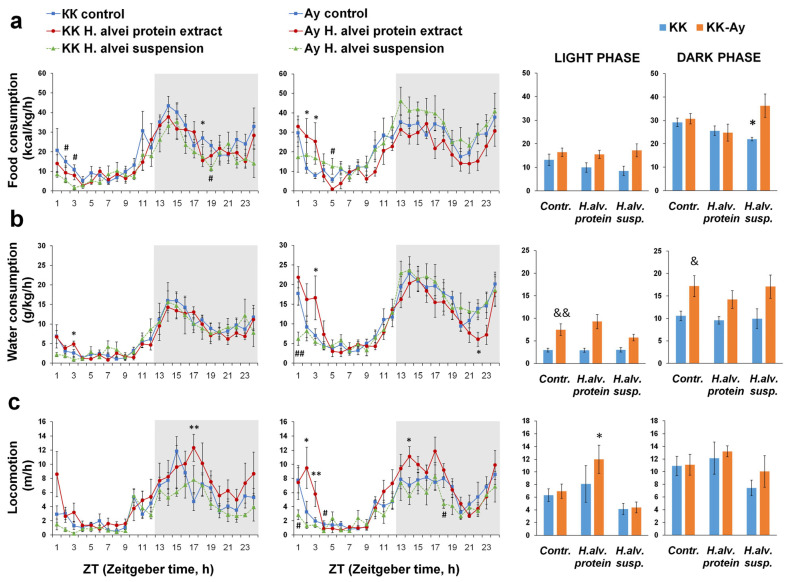
The effect of *H. alvei* protein extract (*H. alvei protein*) or *H. alvei* bacterial suspension (*H. alvei* susp.) on food (**a**) and water (**b**) consumption and locomotor activity (**c**) in KK.Cg-a/a (KK) and KK.Cg-Ay/a (Agouti yellow; KK-Ay) mice. Control groups received saline. The graphs on the left show the reaction rates in the light (unshaded background) and dark (shaded area) phases. The right panels show the averaged measurements in the light and dark phases. Comparisons using one-way ANOVA with Fisher LSD post hoc test: * *p* < 0.05, ** *p* < 0.01—control vs. protein; # *p* < 0.05, *## p*<0.01—control vs. bacteria; & *p* < 0.05, && *p* < 0.01—between control groups of KK and KK-Ay. Number of animals KK/KK-Ay per group: controls—7/10, *H. alvei* protein extract—6/6, *H. alvei* suspension—4/8.

An assessment of energy balance was conducted on days 18–20 of the treatment period using indirect calorimetry, along with continuous measurements of food and water intake, and locomotory activity. No difference in food consumption normalized to body weight was found between the control groups; however, treatment of KK mice with the suspension of live bacteria resulted in a reduction in food consumption during the dark period (Figure 3a). Water intake was higher in the KK-Ay than in KK sub-strain throughout the day. Probiotic treatment did not affect differences in water consumption either within or between sub-strains (Figure 3b).

Although physical activity levels did not differ between the control groups, KK-Ay mice receiving the bacterial protein extract showed increased daytime motor activity compared to the control group of the same sub-strain. No effect of probiotic treatment on physical activity was found in KK mice (Figure 3c).

**Figure 4:**
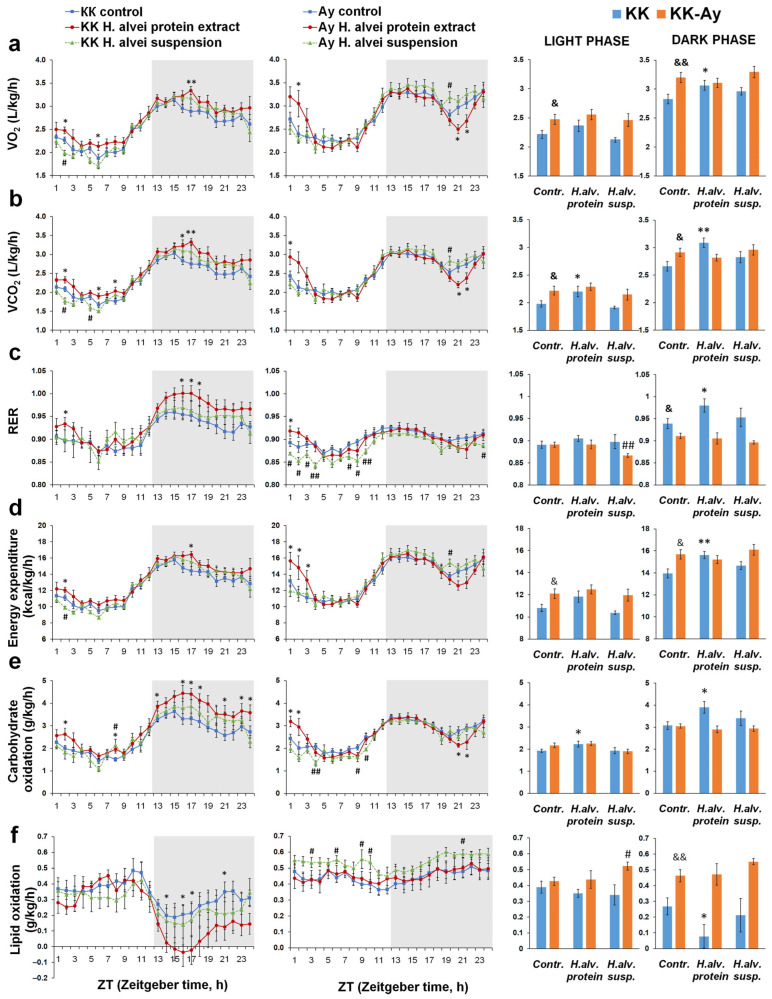
The effect of intragastric administration of *H. alvei* protein extract (*H. alvei protein*) or *H. alvei* bacterial suspension (*H. alvei* susp.) on respiratory exchange and energy balance in KK.Cg-a/a (KK) and KK.Cg-Ay/a (Agouti yellow; KK-Ay) mice: (**a**) oxygen consumption, (**b**) CO_2_ production, (**c**) respiratory exchange ratio (RER), (**d**) energy expenditure, (**e**) carbohydrate oxidation, and (**f**) lipid oxidation. The control group received saline solution. The graphs on the left show changes in metabolic rates during light (unshaded background) and dark (shaded area) periods of the day. The graphs on the right show average measurements during light and darkness. Comparisons using one-way ANOVA with Fisher LSD post hoc test: * *p* < 0.05, ** *p* < 0.01—control vs. protein; # *p* < 0.05, ## *p* < 0.01—control vs. bacteria; & *p* < 0.05, && *p* < 0.01—between control groups of KK and KK-Ay. Number of animals KK/KK-Ay per group: controls—7/11, *H. alvei* protein extract—6/6, *H. alvei* suspension—4/8.

Gas exchange measurements in control groups showed higher O_2_ consumption and CO_2_ production in KK-Ay mice compared to KK mice during both light and dark periods (Figure 4a,b). These differences in KK-Ay were associated with an increased energy expenditure throughout the day (Figure 4d). Respiratory exchange ratio was lower in KK-Ay controls at night (Figure 4c), indicating a shift in metabolism toward lipid oxidation. Indeed, the calculated lipid oxidation during the dark period was greater in the KK-Ay control group than in the KK control mice (Figure 4f). No significant differences in the carbohydrate oxidation between the control groups were observed (Figure 4c,e).

Supplementation with *H. alvei* bacterial protein extract resulted in a marked increase in O_2_ consumption during the dark phase and CO_2_ production during the light and dark phases in KK mice (Figure 4a,b), which was reflected in an increase in both RER and energy expenditure during the dark phase (Figure 4c,d). Moreover, KK mice receiving bacterial protein extract displayed a marked increase in carbohydrate oxidation during both phases and lower lipid oxidation during the dark period (Figure 4e,f). Treatment of KK mice with *H. alvei* bacterial suspension did not produce any significant shift in any of these metabolic parameters.

In contrast to the KK sub-strain, in KK-Ay mice, after treatment with the suspension of live bacteria, the RER was significantly reduced throughout the day and lipid oxidation increased during the light period (Figure 4c,f).

### 2.4. Effect of Probiotics on Glucose Tolerance and Insulin Resistance

In the glucose tolerance test following a six-hour period of fasting, the control group of KK-Ay mice demonstrated higher baseline plasma glucose concentrations than the matching KK group (16.83 ± 0.60 vs. 13.41 ± 0.51, *p* < 0.001, Student’s test).

**Figure 5:**
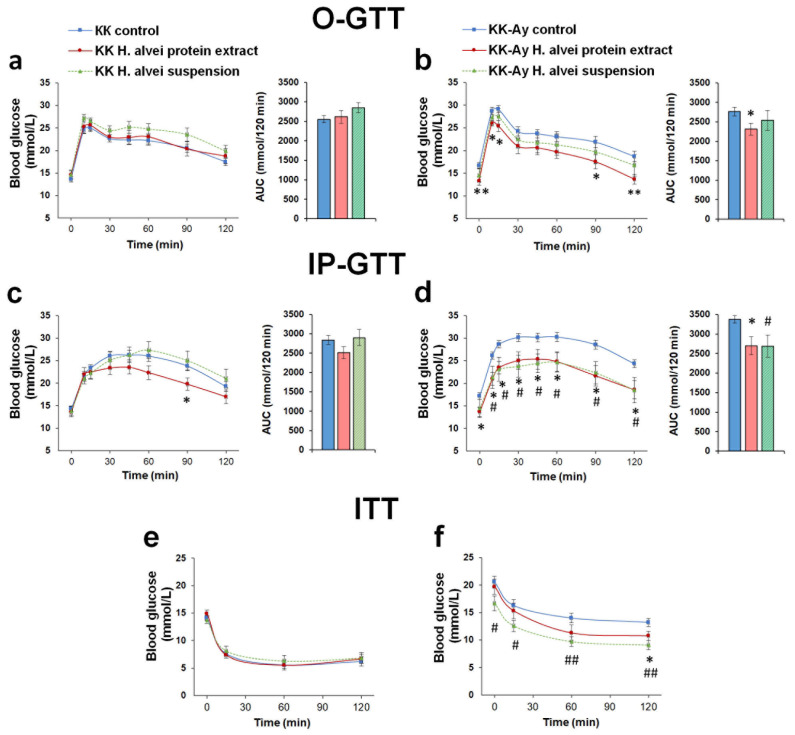
The effect of intragastric administration of *H. alvei* protein extract or *H. alvei* bacterial suspension on glucose (**a**–**d**) and insulin (**e**,**f**) tolerance in the KK.Cg-a/a (KK) or KK.Cg-Ay/a (KK-Ay) mouse sub-strains as compared to controls receiving saline. (**a**–**d**) demonstrate the change in plasma glucose concentration following oral (O) or IP glucose load. The area under the concentration curve (AUC) is shown as a histogram in the panel. Glucose tolerance was evaluated following 6 h of food deprivation; animals were administrated 1.0 g/kg glucose. Panels e and f illustrate the alteration in plasma glucose concentration subsequent to the intraperitoneal administration of insulin (2.0 U/kg) to non-fasted mice. Comparisons using two-way ANOVA with repeated measurements or one-way ANOVA (for AUC’s data) with Fisher LSD post hoc test: * *p* < 0.05, ** *p* < 0.01—control vs. protein; # *p* < 0.05, ## *p* < 0.01—control vs. bacteria. The number of animals per group: KK controls—11~12; KK with H. alvei protein extract—5; KK and H. alvei suspension—6; KK-Ay controls—13~14; KK-Ay with H. alvei protein extract—8~9; KK-Ay with H. alvei suspension—7~8.

In the KK mice, O-GTT did not reveal any probiotic effects: probiotic effect F(2, 20) = 1.51, *p* > 0.24; time effect F(7, 140) = 114.79, *p* < 0.00001; probiotic × time F(14, 140) = 0.64, *p* > 0.82. The comparison of AUCs corroborates the absence of an effect in the O-GTT (F(2, 27) = 1.79, *p* > 0.18; Figure 5a). However, O-GTT in KK-Ay mice showed that the group treated with *H. alvei* bacterial protein exhibited lower plasma glucose levels than the control group both before and 15, 90 and 120 min after glucose loading (Figure 5b). This effect was confirmed by comparisons between AUCs (F(1, 20) = 5.37, *p* < 0.05, Figure 5b).

The provision of KK mice with probiotics resulted in visible improvement in plasma glucose disposal after IP glucose load; although this was not confirmed by two-way ANOVA: probiotic effect F(2, 17) = 1.01, *p* > 0.38; time effect F(7, 119) = 93.42, *p* < 0.00001; probiotic × time F(14, 119) = 2.86, *p* < 0.001 (Figure 5c). However, the statistical significance of the effect of bacterial protein extract was shown by one-way ANOVA (*p* < 0.05). Comparisons between AUC values did not demonstrate significant differences in IP-GTT (F(1, 12) = 2.63, *p* > 0.13; Figure 5c).

By analyzing the percentage of changes in blood glucose from the pre-test baseline levels, it was evident that improved glucose tolerance in bacterial protein-treated KK mice in the IP-GTT was due to the reduced amplitude of the response to glucose administration (Supplemental Figure S1b). On the contrary, improved glucose tolerance in probiotic-treated KK-Ay mice, in both O-GTT and IP-GTT, was due to the reduced pre-test baseline level of glucose in these groups (Figure S1d,e).

In both mouse strains, the administration of insulin (2.0 U/kg, IP) caused a rapid decrease in plasma glucose level, whose magnitude reflected the tissue insulin resistance (Figure 5e,f). Treatment with probiotics did not influence insulin resistance in KK mice as confirmed by the two-way ANOVA: effect of probiotic F(2, 19) = 0.14, *p* > 0.86; effect of time F(3, 57) = 152.95, *p* < 0.00001; probiotic × time F(6, 57) = 0.66, *p* > 0.68 (Figure 5e). Probiotics had an effect in the KK-Ay strain, showing that the group treated with live bacterial suspension had improved insulin resistance as compared to the control group throughout the test, while the mice treated with bacterial protein exhibited a significant improvement by 120 min of the experiment (Figure 5f). Comparison of percentage changes in glucose levels in the ITT confirmed an improved tolerance to insulin administration in both *H. alvei*-treated groups of KK-Ay mice (Figure S1f): effect of probiotic F(2, 27) = 4.14, *p* < 0.027; effect of time F(3, 81) = 166.66, *p* < 0.00001; probiotic × time F(6, 81) = 2.094, *p* < 0.063. Post hoc analysis revealed significant differences between both experimental groups and control at 60 and 120 min after injection (Figure S1f).

### 2.5. Effect of Probiotics on mRNA Expression of Metabolism-Regulating Genes in the Hypothalamus

**Figure 6:**
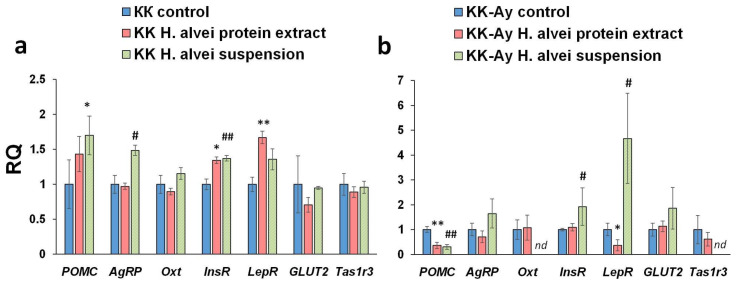
Expression of mRNA of hypothalamic metabolism-regulating genes in mouse sub-strains KK.Cg-a/a (KK, (**a**)) and KK.Cg-Ay/a (KK-Ay, (**b**)) treated via gavage with *H. alvei* protein extract or *H. alvei* bacterial suspension for 21 days. The control group received saline. Data is presented as a relative quantity (RQ = 2^−ddCt^) to the control group. POMC—proopiomelanocortin, AgRP—Agouti-related peptide, Oxt—oxytocin, InsR—insulin receptor, LepR—leptin receptor, GLUT2—glucose transporter 2, *Tas1r3*—type 1 taste receptor subtype 3. Comparisons with control group were performed using Student’s *t*-test: * *p* < 0.05, ** *p* < 0.01—control vs. protein; # *p* < 0.05, ## *p* < 0.01—control vs. bacteria. Number of animals KK/KK-Ay per group: control—5/7, *H. alvei* protein extract—5/3~8, *H. alvei* suspension—4/3.

Treatment with *H. alvei*-based preparations had significant effect on the mRNA expression levels of a number of hypothalamic genes involved in metabolic regulation. As shown in Figure 6a, administration of bacterial protein extract to KK mice resulted in increased mRNA expression of both insulin and leptin receptors (InsR and LepR, respectively), whereas supplementation with bacterial protein extract inhibited the expression of LepR mRNA in the KK-Ay strain (Figure 6b). The live bacteria suspension also potentiated the expression of InsR in KK mice and both InsR and LepR in KK-Ay mice. In KK mice, the expression of POMC and AgRP mRNA was stimulated after treatment with the live bacteria suspension, while in KK-Ay group, POMC mRNA synthesis was suppressed by both *H. alvei* formulations. Expression of oxytocin (Oxt), glucose transporter 2 (GLUT2) and sweet taste receptor subunit *Tas1r3* mRNA was not affected by probiotics in either strain of mice (Figure 6a,b).

### 2.6. Melanocortin Receptor Type 4 (MC4R) Activation In Vitro

**Figure 7:**
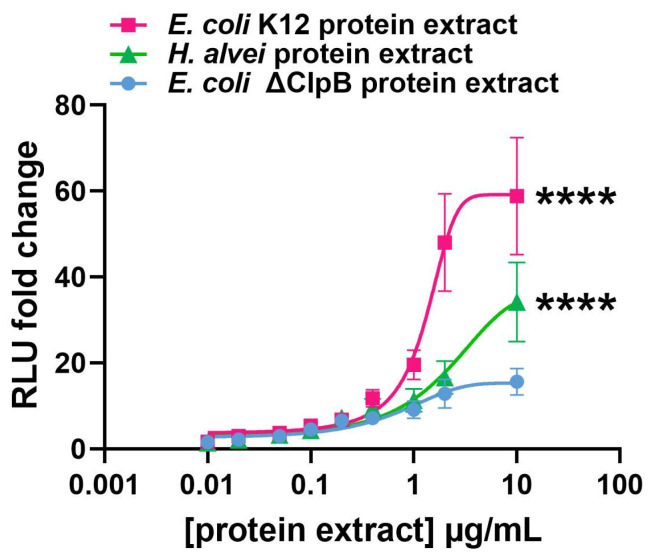
Dose–response of the MC4R in vitro activation by bacterial protein extracts using TANGO assay of β-arrestin recruitment measured as fold relative luminescence units (RLU) change over baseline. Bacterial protein extracts from *E. coli* K12 (*n* = 10), *E. coli* K12 ΔClpB (*n* = 10), and *H. alvei* (*n* = 10) were used. Comparisons were made with two-way ANOVA followed by the Bonferroni post-test vs. 0.01 mg/mL, **** *p* < 0.0001.

A dose-dependent increase in luminescence reads from the background level was observed for all three bacterial protein extracts which showed different capacities of the MC4R activation (Figure 7). As such, the *E. coli* K12 extract induced the strongest MC4R activation close to 60-fold at the maximal 10 µg/mL protein concentration (2-way ANOVA, Bonferroni post-test vs. 0.01 mg/mL, *p* < 0.0001). In contrast, the protein extract of *E. coli* K12 ΔClpB induced a non-significant response. The *H. alvei* extract also significantly stimulated MC4R, inducing about 40-fold activation at a concentration of 10 µg/mL.

The potencies of MC4R activation between 3 bacterial extracts were statistically compared at their maximal concentrations and as a total impact of different concentrations. Higher activation of MC4R by *E. coli* K12 rather than by ClpB-deficient strain *E. coli* K12 ΔClpB was found using both analyses, validating ClpB as the main protein in *E. coli* K12 underlying the MC4R activation.

## 3. Discussion

The impact of gut microbiota on the regulation of host metabolism is multifaceted, involving several molecular pathways [29]. In particular, gut microbiota may be linked to host neuroendocrine regulation of energy balance through gut bacteria-derived metabolites. Among them, bacterial ClpB protein produced by the *Enterobacteriaceae* and *Hafniaceae* families appears of interest due to its structural homology with the anorexigenic α-MSH [4,6]. Indeed, activation by ClpB of both intestinal satiety hormone PYY and hypothalamic POMC neurons [12,13] justify it as a microbiota-derived anorexigenic factor, which may act on the same targets as the endogenous anorexigenic signals α-MSH and leptin [9,11,30,31]. Thus, the primary objective of the present study was to verify the contribution of MCRs to the regulation of metabolic responses to intragastric administration of a suspension of live *H. alvei* bacteria or *H. alvei* bacterial protein extract. However, it is important to acknowledge that this does not preclude the possible involvement of other non-MCR-mediated mechanisms of action of *H. alvei*-based preparations, which are beyond the scope of this discussion.

To address the objectives of the study, the experiments were performed with closely related mouse sub-strains KK.Cg-a/a and KK.Cg-Ay/a, which are established models for diabetes and obesity research [32]. The ectopic expression of the Ay allele in KK-Ay mice results in the overproduction of the Agouti protein, a competitive inhibitor of the melanocortin receptors MC1R, MC3R, and MC4R [21,22]. Consequently, it can be assumed that in the presence of the Ay allele, the responses to α-MSH or its mimetics should be abolished or diminished. Thus, if the primary influence of *H. alvei* on host metabolism is achieved through MCRs, the KK-Ay sub-strain should exhibit reduced responses to the supplementation of this probiotic as compared to the parental KK strain having functional MCRs.

Two types of *H. alvei* probiotic preparations were used: a live bacterial suspension and a bacterial protein extract. While bacterial proteins may act in the small intestine before they are digested and absorbed, live bacteria may reach the large intestine, where they can transiently persist and release their metabolites interacting with the intestinal epithelium and enteroendocrine cells. Thus, by using a *H. alvei* protein extract, it is possible that higher concentration of bacterial proteins can reach the host targets. Potential limitations of the study relate to the use of saline as a control, but not protein-matched control. However, this is entirely consistent with the objectives of the study to determine the extent to which the preparation’s action is related to melanocortin signaling, for which the saline control used is quite suitable.

A more pronounced obese and diabetic traits observed in the KK-Ay mice even under a low-calorie diet confirmed the contribution of deficiency of melanocortin signaling in the obese phenotype. As such, comparison of the KK and KK-Ay control groups (saline gavage) demonstrated that the expression of the Ay allele was associated with alterations in metabolic parameters that are also characteristic of impaired melanocortin regulation, namely increased body weight, relative liver weight, blood glucose and liver glycogen (Figure 1a,c and Figure 2a,b). On the other hand, under a low-calorie diet, no differences were found in other obesity signs such as insulin, leptin and body fat levels between the control groups of both sub-strains. Moreover, untreated KK-Ay mice showed a slight increase in systemic energy expenditure, as well as increased lipid oxidation, primarily during the dark period (Figure 4d,f). Why such anti-obesity metabolic parameters were not altered in the more severe obesity phenotype is unclear; we may speculate on the role of MC3R, which may remain partially functional in KK-Ay mice. A recent study has identified systemic anti-obesity effects of MC3R acting in the liver [33].

The influence of melanocortin signaling on fat mass can be through the modulation of the sympathetic and parasympathetic activity of nerve fibers innervating the adipose tissue [34]. In addition, it may involve the MCRs expressed by the adipocytes, MC3-4R and the fifth type of melanocortin receptor (MC5R), stimulating adipocyte lipolysis [35,36,37].

The results of the present study generally confirmed our hypothesis that the presence of fully functional MCRs is required for a number of beneficial anti-obesity effects of *H. alvei*. However, while such conclusion is valid for the energy metabolism and food intake, some antidiabetic effects of probiotic treatment were also present in KK-Ay mice, which we discuss below in more detail.

Importantly, we demonstrated that *H. alvei* protein extract has the capacity to enhance energy metabolism in KK but not in KK-Ay mice. As such, increased O_2_ consumption, CO_2_ output, carbohydrate oxidation, elevated RER and energy expenditure and locomotion were observed in KK mice during the dark phase, while in the light phase a non-significant tendency of increase was observed (Figure 3c and Figure 4a–e). Such results indicate that the metabolic effects of *H. alvei* protein preparation depend on the functional presence of MCRs and are influenced by the circadian rhythm, a phenomenon which was earlier shown for the microbiota–host interactions [38]. Moreover, *H. alvei* suspension was effective in decreasing food intake in KK but not in KK-Ay mice (Figure 3a), indicating a role of MCRs in mediating an anorexigenic effect of *H. alvei*. In spite of reduced food intake in *H. alvei*-treated mice, we did not observe a decrease in body weight, which was probably due to a relatively short time of treatment. Nevertheless, a significant reduction in total fat mass was present in KK mice receiving *H. alvei* protein, demonstrating its more pronounced anti-obesity effect than the live bacteria *H. alvei* suspension in concordance with the activation of metabolism in these mice (Figure 1b).

Taken together, these results support the role of MCRs in mediating the anti-obesity effects of *H. alvei*. Since such effects include food intake, locomotion, and systemic energy metabolism, i.e., functions regulated primarily in the brain, it is likely that KK-Ay mutation prevents the access of *H. alvei*-derived α-MSH-like ligands to central MCRs underlying the absence of *H. alvei* anti-obesity metabolic and behavioral effects in this mouse strain.

Surprisingly, exposure to both protein extract and suspension of *H. alvei* also reduced total fat mass in KK-Ay mice (Figure 1b). These changes probably implicate a different regulatory pathway than in KK mice, e.g., increased lipid oxidation. Since the Ay mutation does not equally result in inhibition of all types of MCRs, in particular, retaining some sensitivity of MC3R and MC5R, it is possible that *H. alvei*-derived signals, such as ClpB, may directly stimulate lipolysis, re-esterification and fatty acid oxidation in adipocytes [36]. Such possibility is supported by recent demonstration of MC4R activation by ClpB [9].

There is now substantial evidence that live *H. alvei* bacteria, when administered as a probiotic dietary supplement, can reduce blood glucose levels in healthy overweight mice and humans [3,5]. Moreover, improved glucose tolerance was observed in non-diabetic lean C57B6/J mice following the administration of the *H. alvei* protein extract or ClpB protein [14]. The hypoglycemic effect of *H. alvei* and ClpB may be mediated by the action of both central and peripheral melanocortin signaling, which have been identified as key regulators of glucose and insulin homeostasis [39]. The present study, conducted on diabetic strains of mice maintained on a low-calorie diet, supported the ability of the *H. alvei*-based preparations to improve glucose tolerance. The treatment of the KK strain with the bacterial protein extract resulted in an enhancement of glucose clearance in IP-GTT, yet no discernible effect was observed in O-GTT (Figure 5a,c). Different IP- and O-GTT results were also found in non-diabetic mice [14], suggesting that *H. alvei*-derived proteins may primarily affect post-absorptive glucose-related glycemic regulatory mechanisms. These mechanisms may involve parasympathetic cholinergic fibers innervating the pancreas and expressing MC4Rs [40,41].

Indeed, our study revealed that *H. alvei* extract is able to stimulate MC4R. Although the α-MSH-like epitope of ClpB is the same in *H. alvei* and *E. coli*, the full proteins have about 80% homology [4]. Since the entire ClpB protein is needed for the MC4R activation [9], different conformational structures of *E. coli* and *H. alvei* ClpB may underlie their different potencies of MC4R activation; alternatively, these bacterial preparations may have different ClpB concentrations.

Oral treatment with *H. alvei* protein extract (O-GTT) improved glucose tolerance in KK-Ay mice. In IP-GTT, both *H. alvei* protein extract and live bacteria significantly enhanced blood glucose clearance (Figure 5b,d). These effects were distinct from those in KK mice, since the improvement was due to a lower baseline glucose level in treated groups of KK-Ay, as demonstrated by comparing the percentage of changes in blood glucose levels from the pre-test baseline levels (Figure S1). In KK-Ay mice, *H. alvei*-derived ClpB in the intestinal lumen was likely able to activate the secretion of incretin hormones before glucose load. Improved insulin resistance found in the KK-Ay mice treated with live bacteria further supports the contribution of the incretin effect (Figure 5f). Moreover, reduced liver glycogen content in KK-Ay mice (Figure 2b) may reflect a long-term improvement of tissue glucose utilization. We can also speculate that the novelty of the cage environment during the preparation of these assays contributed to the decrease in glucose levels in ClpB-treated mice, likely through the activation of the endogenous melanocortin system, known for its role in novelty acquisition [42].

The aforementioned results indicate that the Ay mutation has a diminishing effect on the probiotics’ capability to reduce food intake and carbohydrate oxidation, yet it does not prevent their beneficial effect on glucose tolerance and lipolysis. These results can be interpreted as indication for the ability of the probiotic-derived proteins to compete with the Agouti protein at some peripheral targets, e.g., gut, liver, and adipose tissue, thereby improving the Agouti-dependent obesity phenotype. Conversely, the central targets involved in appetite regulation and energy metabolism are presumably less susceptible to such competitive interactions. It is relevant to reiterate that the Ay mutation causing abundant expression of ASIP does not inhibit all types of melanocortin receptors. ASIP shows weak affinity for mouse MC3R [22]. In addition, the Ay mutation has almost no effect on the interaction of α-MSH with MC5R, which is involved in mediating glucose uptake by skeletal muscles [35]. The above-described MCR-independent beneficial effects of probiotics on lipid and glucose metabolism in KK-Ay mice are of particular interest for their potential relevance for the treatment of some rare monogenic forms of human obesity due to ectopic expression of ASIP [43].

Expression of hypothalamic neuropeptides regulating appetite and energy metabolism is affected by gut microbiota composition [44]. Here, we show that the administration of the bacterial protein extract or live *H. alvei* bacteria resulted in a discernible impact on the mRNA levels of POMC and AgRP in the hypothalamus. A substantial discrepancy was observed in the responses exhibited by the KK and KK-Ay strains. Treatment with suspension of live bacteria upregulated the expression of POMC mRNA in the KK strain (Figure 6a), which is concordant with the decrease in food intake in this group (Figure 3a), but both live bacteria and bacterial protein extract reduced POMC and AgRP expression in the KK-Ay strain (Figure 6b). Therefore, the non-selective competitive inhibition of MCR on the background of the Ay mutation resulted in the silencing of the probiotic’s effect on the expression of both orexigenic (AgRP) and anorexigenic (POMC) hypothalamic neuropeptides. It is possible that such an effect on AgRP was secondary to the *H. alvei* induction of gut satiety hormones, whose anorexigenic effect was nevertheless silenced by arcuate neurons by downstream MC4R inhibition.

The insulin and leptin receptors activate the POMC neurons and inhibit the AgRP neurons in the hypothalamus [45,46]. Our study shows that *H. alvei*-based probiotic preparations increase the expression of LepR and InsR mRNA in the hypothalamus. The *H. alvei* protein extract increased the expression of InsR and LepR mRNA in the KK mouse strain, but not in the KK-Ay strain, suggesting that MCRs are involved in the response (Figure 6). Treatment with a suspension of live bacteria increased the expression of LepR and InsR mRNA in the hypothalamus of both strains of mice with almost equal efficacy, suggesting that this pathway of probiotic action may be indirect.

## 4. Materials and Methods

The study was conducted in accordance with the Declaration of Helsinki and approved by the Animal Care and Use Committee at the Pavlov Institute of Physiology (Animal Welfare Assurance #A5952-01), approval No. 03/15, dated 15 March 2022.

### 4.1. Animals

Experiments were performed on 90–140-day-old male mice bred by cross-mating black female KK.Cg-a/a and yellow male KK.Cg-Ay/a mice from the Pavlov Institute breeding colony. The founders of the sub-strains were obtained from The Jackson Laboratory (Bar Harbor, ME, USA). Animals were housed in accordance with the “Mouse room conditions” Jackson Laboratory guidelines (www.jax.org/jax-mice-and-services, accessed on 22 March 2021), four per cage, unless otherwise specified, in standard polycarbonate cages on wood shavings bedding in a temperature and humidity-controlled room (22–24 °C, 40–50% humidity) under a 12/12 h light/dark cycle. During the acclimation period and throughout the experiment, mice were fed a maintenance diet (Lbk 120 C-19, JSC “BioPro”, Novosibirsk, Russia), including 58% carbohydrates, 6% fat, 19% protein as well as vitamins and minerals with an energy value of 2.50 kcal/g. Feed and tap water were freely available. A total of 23 mice from KK strain and 33 mice from the KK-Ay/a sub-strain were used in the study.

### 4.2. Bacterial Culture

Live *Hafnia alvei* HA4597 bacteria were obtained from EnteroSatys^®^ caps (TargEDys, Longjumeau, France) and isolated on Luria–Bertani (LB) agar. A single colony was incubated in 10 mL of LB broth overnight at 37 °C with orbital shaking (140 rpm). A fraction of resulting culture (0.2 mL) was diluted at 10^7^ CFU/mL in 200 mL (Erlenmeyer flask) of LB broth and incubated at 37 °C with orbital shaking (140 rpm) for 24 h. After 6 h of incubation, which corresponded to the beginning of the stationary growth phase, which was checked by optical density, the culture was stopped for sample collection. Bacterial abundance was determined by sowing on LB agar; 5 mL of sample contained 3.5 × 10^8^ CFU. Samples were diluted in 0.9% NaCl to a concentration of 4.5 × 10^7^ CFU in 200 µL, a single dose per mouse, similarly to that used previously [4,14]. Aliquots were stored at −20 °C for further use.

### 4.3. Bacterial Protein Extraction

Bacterial protein was extracted according to a previously published protocol [13]. Briefly, samples of *H. alvei* cultures were centrifuged at 3000× *g* for 20 min at 4 °C in Falcon tubes (50 mL) and supernatant was discarded. To wash out secreted compounds, bacterial pellets were resuspended with 0.1 M phosphate-buffered saline (PBS) and centrifuged at 3000× *g* for 20 min at 4 °C, the supernatant was discarded. Washes were repeated 3 times. To promote lysis, the pellets were frozen overnight at −20 °C. Bacterial cells were lysed in 2 mL R2D2 buffer (7 M urea, 2 M thiourea, 2% CHAPS, 0.05% triphosphobutyl, 20 mM dithiothreitol, 0.5% C7BzO) with sonication for 6 min at 23% amplitude (3 s ON/1 s OFF). The addition of thiourea to the lysis buffer promoted better solubilization of the protein [47]. Solubilized proteins were recovered by taking supernatant after centrifugation at 10,000× *g* for 30 min at 4 °C and quantified using Bradford assay. Protein extracts were diluted in saline (5 µg in 200 µL), a single dose per mouse similar to that used previously [14]. Aliquots were stored at −20 °C for further use.

### 4.4. Experimental Design

Prior to the administration of probiotics, mice were kept for seven days within individual cages for acclimation. The probiotic material was administered on a daily basis at 5 p.m. for a duration of 21 days (0–20) by means of intragastric gavage. Mice of both sub-strains were randomly assigned to three experimental groups. One experimental group received a suspension of *H. alvei* live bacteria (4.5 × 10^7^ CFU in 200 µL of saline/mouse), while the other was provided with a total protein extract of *H. alvei* (5 µg/mouse in 200 µL of saline). Control group received 200 µL of saline solution. As shown previously, treatment with live bacteria and bacterial total protein extract was not accompanied by any adverse effects [4,14]. All of the animals completed the study without attrition or adverse events.

**Figure 8:**
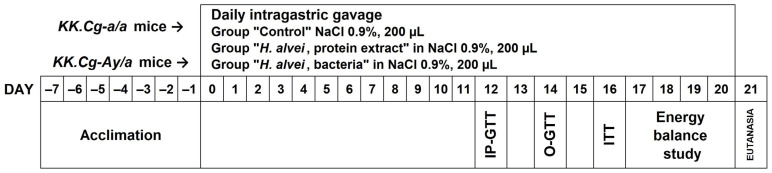
Experimental design. IP-GTT—intraperitoneal glucose tolerance test; O-GTT—intragastric (by oral gavage) glucose tolerance test; ITT—insulin tolerance test.

Intraperitoneal and intragastric (by oral gavage) glucose tolerance tests (IP-GTT and O-GTT, respectively) were performed on days 12 and 14 starting at 1–2 p.m. The insulin tolerance test (ITT) was performed at 12 a.m.–1 p.m. on day 16. After completion of the insulin tolerance test and daily administration of probiotics, animals from each group were placed in separate chambers of the Promethion Core indirect calorimetry system (see below) where they were maintained for 5 days. On day 21, mouse tissues were collected under terminal sedation as described below in detail. All measurements were performed by experimenters blinded to treatment and group allocation. The experimental protocol is schematically illustrated in Figure 8.

### 4.5. Energy Balance Assessment

The open-circuit Promethion Core 2 High-Definition Multiplexed Respirometry System Model CAB-16R (Sable Systems, GmbH, Berlin, Germany) was utilized to synchronously measure oxygen consumption, CO_2_ production, locomotor activity, food and water intake. The system, calibrated according to the manufacturer’s instructions, was operated under artificial light cycles (12 h light/12 h dark) and standard laboratory conditions providing a temperature of 24 °C and humidity of 40–60%. On day 17 of the experiment, mice were placed in individual sealed Promethion chambers with ad libitum access to food and water. A series of measurements were obtained at 10 min intervals. The initial 48 h period was designated as the acclimatization phase, and data from the following three full days (18th–20th) were used for calculations. To estimate energy expenditure and substrate utilization, oxygen consumption (VO_2_) and carbon dioxide production (VCO_2_) were measured as difference at the inlets and outlets of the sealed chambers. The respiratory exchange ratio (RER) indicating substrate utilization was calculated online as VCO_2_/VO_2_. Other metabolic parameters were calculated after the test by conversion of raw data averaged to 1 h slices. Indirect calorimetry data were analyzed separately for the light (8 a.m. to 8 p.m.) and dark (8 p.m. to 8 a.m.) phases of the day.

Energy expenditure was calculated using the Weir formula (3.9 × VO_2_ + 1.1 × VCO_2_) [48] and normalized to body weight. Glucose and lipid oxidation rates were calculated using modified Ferrannini equations [49], assuming that urinary nitrogen excretion is the same between genotypes. Thus, carbohydrate (G) and lipid (L) oxidation rates were estimated using the following equations: G = 4.585 × VCO_2_ − 3.226 × VO_2_ and L = 1.695 × VO_2_ − 1.701 × VCO_2_; both were normalized to body weight [50].

Calorie expenditure per day was calculated by multiplying the amount of food consumed in grams by the caloric content of the diet (2.50 kcal/g) and normalized to gram of the body weight. Locomotor activity was measured by infrared beam breaks on the X and Y axes and converted to distance traveled by the Promethion software (OneClickMacroV.2.50.6).

### 4.6. Body Composition

The body mass of each individual was measured daily for 21 days of treatment with an electronic balance. On day 22, non-fasted mice were terminally sedated with a gas mixture of CO_2_ and O_2_ (50/50 volume %) administered in a gradual fill method [51] and decapitated. Blood was withdrawn into tubes containing EDTA and centrifuged for plasma separation, frozen and kept at −80 °C. Liver, pancreas, and fat were removed and weighed to the nearest 0.001 g. The anterior subcutaneous (interscapular), posterior subcutaneous (dorsolumbar, inguinal, and gluteal), visceral perirenal, visceral mesenteric, and epididymal (gonadal) bilateral fat depots were excised. Brain was taken and the hypothalamus was dissected and kept in IntactRNA formalin-based stabilizing reagent (Evrogen, Moscow, Russia) at −80 °C until further RNA extraction.

### 4.7. Biochemical and ELISA Assays

Glycogen concentration in the liver was measured spectrophotometrically, as described by Danchenko and Chirkin [52]. Non-fasting insulin and leptin assays were performed using ELISA kits CEA448Mu and SEA084Mu (Cloud-Clone Corp., Katy, TX, USA).

### 4.8. Blood Glucose Measurements and Insulin Tolerance Test

Prior to undergoing a glucose tolerance test (GTT), the mice were subjected to a 6 h fast (8:00–14:00) on a clean bedding. A dose of 1.0 g/kg glucose (Sigma Aldrich, Burlington, MA, USA) was administered in aqueous solution via intraperitoneal (IP) or oral (O) injection. In the insulin tolerance test (ITT), non-fasted animals were injected IP with 2.0 U/kg insulin (Actrapid^®^ HM, Novo Nordisk A/S, Bagsvaerd, Denmark). Blood was sampled through a tail incision, and glucose concentrations were measured at 0, 10, 15, 30, 60, 90, and 120 min after glucose administration or at 0, 15, 60, and 120 min after insulin administration. For each time point, two or three repeated glucose concentration measurements were performed using a handheld glucose meter, the Contour Plus One (Ascensia Diabetes Care Holdings AG, Basel, Switzerland). Glucose concentration in non-fasted mice was measured in the middle of the light period via a tail incision just before euthanasia.

### 4.9. Real-Time Quantitative Reverse Transcription Polymerase Chain Reaction (RT-qPCR)

Total RNA was isolated from the hypothalamic tissue using a TRIzol analog (ExtractRNA Reagent, Evrogen, Moscow, Russia) following the manufacturer’s protocol. Then the cDNA was synthesized from 2 μg of RNA using the MMLV reverse transcription kit (Evrogen, Moscow, Russia) and the oligo (dT) oligodeoxynucleotide primers. PCR amplification was performed using a mixture containing 10 ng of the RT product, 400 pM of forward and reverse primers, and the qPCRmix-HS SYBR master mix (Evrogen, Moscow, Russia), with a reaction volume of 10 μL. The amplified signals were continuously detected by the Applied Biosystems QuantStudio^TM^ 5 Real-Time PCR System (Thermo Fisher Scientific Inc., Life technologies Holdings Pte. Ltd, Singapore). The following PCR amplification protocol was used: (i) initial denaturation at 95 °C for 5 min; (ii) 40 cycles of a 3-step amplification and quantification loop (95 °C for 15 s, 57–61 °C for 15 s, and 72 °C for 15 s; data were collected during this step); and (iii) the Melt Curve stage to check for one peak and no primer dimer formation in each reaction containing the template. The primer list can be found in Table 1.

The collected data were analyzed using the delta-delta-Ct method (https://toptipbio.com/delta-delta-ct-pcr, accessed on 16 January 2023). Beta-actin (ActB) and beta-2-microglobulin (B2M) genes were used as reference house-keeping genes. The data are presented as fold levels relative to the geometric mean of expression in the control group of mice.

### 4.10. In Vitro Melanocortin Receptor 4 (MC4R) Activation Assay

Free-cell cultures of *E. coli* K12 (from Rouen University laboratory collection), *E. coli* K12 ΔClpB kindly provided by Axel Mogk [53], and *H. alvei* H4597 (EnteroSatys^®^ caps, TargEDys, Longjumeau, France) were performed in 250 mL Erlenmeyer flasks containing 50 mL of Luria–Bertani broth (LB, BD Difco, New Jersey, NJ, USA). Flasks were inoculated with fresh overnight preculture and incubated for 24 h at 37 °C under shaking 140 rpm. For the ΔClpB strain, kanamycin and spectinomycin were added at a final concentration of 30 µg/mL and 50 µg/mL, respectively. Bacteria were recovered by centrifugation (5000× *g*, 30 min, 4 °C, 5430/5430 R centrifuge, Eppendorf). The pellet was washed three times with 10 mL of Dulbeco’s phosphate-buffered saline (DPBS, Sigma-Aldrich, Merck Life Science, Darmstadt, Germany). The pellet was freeze/thawed (−80 °C/37 °C) and resuspended in a denaturing buffer named R2D2 (7 M urea, 2 M thiourea, 2 mM tri-N-butylphosphine (TBP), 20 mM dithiothreitol (DTT), 0.5% (*w*/*v*) 3-(4-heptyl)phenyl-3-hydroxypropyl) dimethylammoniopropanesulfonate (C7BzO), 2% (*w*/*v*) 3-((3-cholamidopropyl) dimethylammonio)-1-propanesulfonate hydrate (CHAPS)). The bacterial suspension was sonicated twice on ice (3 s ON/1 s OFF, amplitude 24%, 6 min) then the lysate was centrifuged (12,000× *g*, 30 min, 4 °C) and the supernatant was ultra-centrifugated (40,000× *g*, 45 min, 4 °C). Protein concentration was evaluated on the final supernatant using the Bradford assay (Bio-Rad, Hercules, CA, USA).

HTLA cells were maintained in DMEM supplemented with 10% FBS, 2 μg/mL puromycin, and 100 μg/mL hygromycin B in a humidified atmosphere at 37 °C in 5% CO_2_. Cells were stably transfected with 2 μg Tango-human *mc4r* construct using lipofectamine 2000 reagent and selected with 500 μg/mL of geneticin. On day 1, cells were plated on a white 96-well plate at the rate of 2 × 10^4^ cells per well in 90 μL of supplemented DMEM. Six hours later, different concentrations of protein extract from *E. coli* K12, *E. coli* K12 ΔClpB, and *H. alvei* were added at the rate of 10 μL/well, in duplicate. To measure constitutive basal activity, no protein extract was added. On day 2, the medium and drugs were slowly removed from the wells (aspiration), and 50 μL of 1/20X Bright-Glo^TM^ luciferase substrate solution (Promega Corporation, Madison, WI, USA) was added to each well. After 2 min, luminescence was counted with an Infinite F200Pro plate reader (Tecan, Mannedorf, Switzerland). Relative luminescence unit (RLU) values were exported into Excel sheets.

### 4.11. Statistical Analyses

The data from all experiments are presented as mean ± SE to reflect the precision of the estimated mean rather than the biological variability among individual animals [54] with *p*-values < 0.05 considered as significant. Statistical analyses were performed primarily using Statistica 12.0 software (RRID:SCR_014213; StatSoft, Tulsa, OK, USA), and all graphs were generated using Excel (Microsoft Corp.). Baseline glucose, plasma hormone levels, and body composition parameters were compared using an unpaired, two-tailed Student’s *t*-test after checking for normal distribution. Indirect calorimetry data were compared using a one-way analysis of variance (ANOVA), and glucose tolerance test (GTT) and intravenous glucose tolerance test (ITT) data were compared using a one- or two-way ANOVA with repeated measures. For two-way ANOVA, time was considered as within-subject factor, and effect of gavage as a between-subject factor. Post hoc paired comparisons were made using Fisher’s least significant difference (LSD) test. For GTT data, the total area under the curve (AUC) was calculated according to the trapezoidal rule. RT-PCR data are presented as a relative quantity (RQ = 2^−ddCt^) to the control group and were compared using two-tailed Student’s *t*-test. MC4R activation data were analyzed using GraphPad Prism 9 (GraphPad Software Inc., San Diego, CA, USA) and compared using two-way analysis of variance (ANOVA) followed by Bonferroni’s post hoc test.

## 5. Conclusions

This study on closely related mouse sub-strains that differ in MCR signaling—KK.Cg-a/a and KK.Cg-Ay/a—known as genetic models of obesity and D2T, revealed that the body weight lowering and glucose tolerance effects of the probiotic bacterium *Hafnia alvei* HA4597 are mediated by MCRs. Treatment of KK mice with *H. alvei* protein extract stimulated total energy expenditure and carbohydrate oxidation but reduced lipid oxidation, leptin levels, and pancreatic weight. The increased expression of insulin and leptin receptors in the hypothalamus of KK mice may contribute to these beneficial effects. Furthermore, in vitro analysis showed that the melanocortin receptor type 4 is a target for the *H. alvei* protein extract, which increases receptor activity.

Additionally, it was established that several beneficial effects of *H. alvei*-based probiotics on energy metabolism were sustained in the Agouti-mutated strain such as reduction in body fat with certain effects being more marked than in KK mice, including a reduction in liver glycogen and improved glucose tolerance. This indicates the potential significance of *H. alvei* protein extract in the management of some rare monogenic forms of human obesity, in particular those caused by ectopic expression of ASIP. Taken together with the demonstration of the capacity of *H. alvei* protein extract to activate MC4R, our proof-of-concept study suggests that both bacterial suspension and bacterial protein extract of the *H. alvei* 4597 strain may represent an effective addition to the treatment of diabetes and obesity in D2T patients.

## Figures and Tables

**Table 1:** PCR primer sequences.

Gene (Abbreviation)	Forward Primer (5′ to 3′)	Reverse Primer (5′ to 3′)	T_ann_ (f/r)	Product Length, b.p.
Beta-2-microglobulin (B2M)	ACAGTTCCACCCGCCT CACATT	TAGAAAGACCAGTCCTTGCT GAAG	60/60	105
Beta-actin (ActB)	CATTGCTGACAGGATG CAGAAGG	TGCTGGAAGGTGGACAGTG AGG	60/60	138
Type 1 taste receptor subtype 3 (Tas1r3)	ATAGTGCCAGCATGGA TCGG	CCTTCCCGGCCATAGTC ATC	60/60	166
Leptin receptor (LepR)	GTGATATTTGGTCCTCT TCTTCTGG	CTGCTTTCAGGGTCTGG TGT	59.2/59.9	140
Insulin receptor (InsR)	TTGGAGCCTTTCTAA ATGTTTCCCTGT	AATACAGTTCAGCTCTTC CTGCCAATC	63/63.4	129
Pro-opiomelanocortin (POMC)	CAGTGCCAGGACCTCA CC	CAGCGAGAGGTCGAG TTTG	59/59	72
Agouti-related peptide (AgRP)	CCCAGAGTTCCCAGGTCT AAGTCT	CACCTCCGCCAAAGC TTCT	61/61	100
Oxytocin (Oxt)	GACCTGGATATGCGCAA GTGT	GAAGCAGCCCAGCTC GTC	60/61	96
Facilitated glucose transporter, member 2 (GLUT2)	GCCTAAAACCGAGGAA CCGAC	TCGCACACCGAGGAAG GAAT	61.3/61.5	72

T_ann_ (f/r)—annealing temperature.

## Data Availability

The raw and processed data that support the findings of this study are available from the corresponding author upon reasonable request.

## Supplementary Materials

The following supporting information can be downloaded at https://www.mdpi.com/article/10.3390/ijms27156963/s1.
