# Supplementary material for: Melanocortin Receptor Signaling Modulates Anti-Obesity and Glucose Tolerance Effects of Hafnia alvei 4597 Probiotic Strain in Mice
Source: Int J Mol Sci. 2026 Aug 3;27(15):6963. doi: 10.3390/ijms27156963 (PMC13467215; doi:10.3390/ijms27156963)
Supplement: Supplementary file 1 [file ijms-27-06963-s001.zip › ijms-4401663-supplementary.pdf]

Supplementary Materials

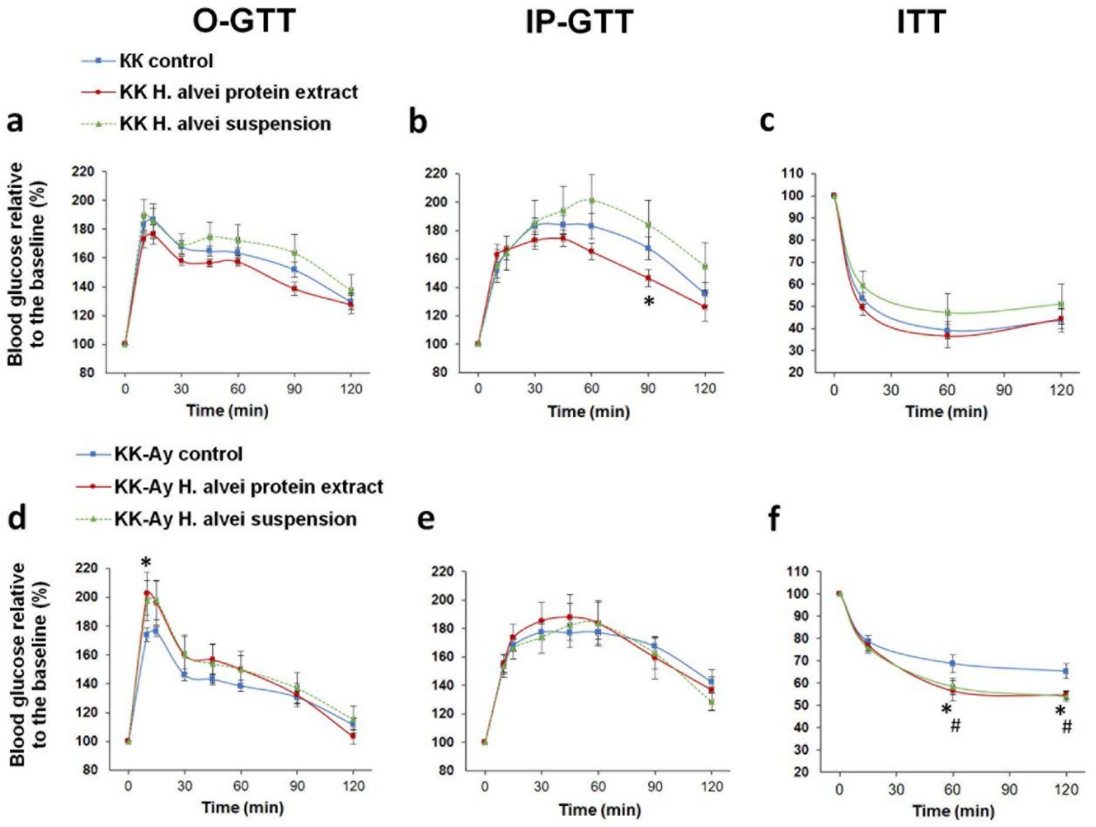

Supplemental Figure S1 The effect of intragastric administration of *H. alvei* protein extract or *H. alvei* bacterial suspension on the glucose (a, b, d, e) and insulin (c, f) tolerance in the KK.Cg-a/a (KK) or KK.Cg-Ay/a (KK-Ay) mouse strains as compared to those of the control groups which received saline. The data normalized to the baseline concentration (before injection). The panels demonstrate the plasma glucose concentration following oral glucose injection (a, d), IP glucose injection (b, e) or after IP insulin injection (c, f). Glucose tolerance was assessed after a 6-hour food deprivation; 1.0 g/kg of glucose was administered to the animals. Insulin (2.0 U/kg) tolerance was checked in non-fasted mice. Post-hoc statistical comparisons were made with Fisher LSD test: * $p<0.05$ – control vs. protein. The number of animals per group: KK controls – 11~12; KK and *H. alvei* protein extract – 5; KK and *H. alvei* suspension – 6; KK-Ay controls – 13~14; KK-Ay with *H. alvei* protein extract – 8~9; KK-Ay with *H. alvei* suspension – 7~8.
